# Graphene Quantum Dot-Mediated Atom-Layer Semiconductor Electrocatalyst for Hydrogen Evolution

**DOI:** 10.1007/s40820-023-01182-7

**Published:** 2023-09-28

**Authors:** Bingjie Hu, Kai Huang, Bijun Tang, Zhendong Lei, Zeming Wang, Huazhang Guo, Cheng Lian, Zheng Liu, Liang Wang

**Affiliations:** 1 Institute of Nanochemistry and Nanobiology, School of Environmental and Chemical Engineering, Shanghai University, 99 Shangda Road, BaoShan District, Shanghai, 200444 People’s Republic of China; 2 State Key Laboratory of Chemical Engineering, Shanghai Engineering Research Center of Hierarchical Nanomaterials, School of Chemistry and Molecular Engineering, East China University of Science and Technology, Shanghai, 200237 People’s Republic of China; 3 School of Materials Science and Engineering, Nanyang Technological University, 50 Nanyang Avenue, Singapore, 639798 Singapore

**Keywords:** Graphene quantum dots, MoS_2_ nanosheets, Atom-layer, Semiconductor electrocatalysts, Hydrogen evolution reaction

## Abstract

The hydrogen evolution reaction performance of semiconducting 2H-phase molybdenum disulfide (2H-MoS_2_) presents a significant hurdle in realizing its full potential applications. Here, we utilize theoretical calculations to predict possible functionalized graphene quantum dots (GQDs), which can enhance HER activity of bulk MoS_2_. Subsequently, we design a functionalized GQD-induced in-situ bottom-up strategy to fabricate near atom-layer 2H-MoS_2_ nanosheets mediated with GQDs (ALQD) by modulating the concentration of electron withdrawing/donating functional groups. Experimental results reveal that the introduction of a series of functionalized GQDs during the synthesis of ALQD plays a crucial role. Notably, the higher the concentration and strength of electron-withdrawing functional groups on GQDs, the thinner and more active the resulting ALQD are. Remarkably, the synthesized near atom-layer ALQD-SO_3_ demonstrate significantly improved HER performance. Our GQD-induced strategy provides a simple and efficient approach for expanding the catalytic application of MoS_2_. Furthermore, it holds substantial potential for developing nanosheets in other transition-metal dichalcogenide materials.

**Supplementary Information:**

The online version contains supplementary material available at 10.1007/s40820-023-01182-7.

## Highlights

- The functional groups on graphene quantum dots (GQDs) for boosting the formation of MoS_2_ nanosheets via theoretical calculations were predicted.
- Near atom-layer-QD@SO_3_ with about 2 nm were synthesized using a functionalized GQD-induced in-situ bottom-up approach.
- Mechanistic insight on the role of functionalized GQDs was elaborated, namely, electron-withdrawing group functionalized GQDs facilitate the formation of nanosheet architectures of MoS_2_ compared to electron-donating group.

**Supplementary Information:**

The online version contains supplementary material available at 10.1007/s40820-023-01182-7.

## Graphical abstract

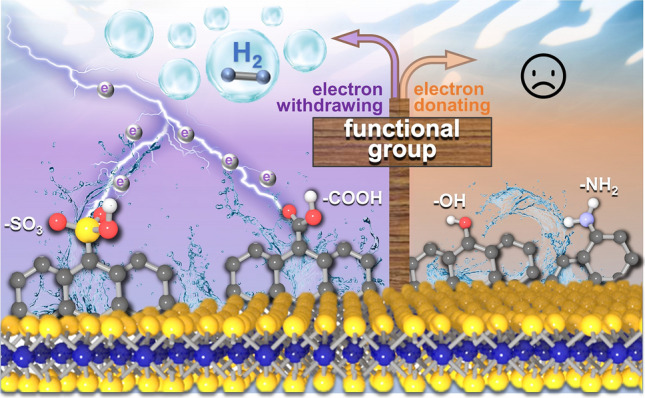

## Introduction

The electrocatalytic production of hydrogen from water represents a promising approach for achieving global carbon neutrality through the utilization of clean energy sources [1–4]. To enable efficient energy conversion, robust and cost-effective electrocatalysts are necessary [4, 5]. Semiconducting 2H-phase molybdenum disulfide (2H-MoS_2_), the most extensively studied transition metal dichalcogenides, has emerged as a leading contender for replacing commercial Pt-based catalysts due to its low cost, earth abundance, and non-toxicity [6, 7]. Unlike bulk MoS_2_, near single-layer 2H-MoS_2_ nanosheets (2H-MoS_2_ NSs) have garnered significant attention due to their high specific surface area and surface activity, rendering them attractive candidates for catalytic hydrogen evolution reaction (HER), electronics, and batteries [8–10]. Nevertheless, the HER capacities of 2H-MoS_2_ NSs are currently limited. Several chemical or physical approaches have been explored to mediate the electronic structure of 2H-MoS_2_ NSs, including light irradiation, energetic ion bombardment, laser ablation, and physical sputtering [4, 6, 11]. However, these methods usually require harsh experimental conditions, such as supercritical hydrothermal, explosive gas atmosphere, and high-risk reagents [12–15], posing risks and limiting practical applications. Therefore, there is a pressing need to develop a method for producing near atom-layer 2H-MoS_2_ under mild conditions to reveal their properties and electrocatalysis applications.

Recently, 2H-MoS_2_ NSs were synthesized using hydrothermal or solvothermal methods. However, their electrocatalytic performance remains suboptimal due to intrinsic poor electronic conductivity. To address this issue, incorporating carbon materials with 2H-MoS_2_ NSs has been explored as an effective strategy to enhance their conductivity [16, 17]. However, the combination of 2H-MoS_2_ NSs with carbon materials leads to irreversible aggregation caused by van der Waals attractions between the sheets. This aggregation reduces the number of exposed active sites, resulting in a decrease in electrocatalytic activity [18]. Graphene quantum dots (GQDs), a novel type of carbon nanomaterials, has attracted significant attention for their unique characteristics, including tunable size, sufficient active sites, and abundant surface functional groups. These features make them highly promising for applications in the electrocatalysis industry [16, 17]. In our previous studies, we successfully utilized GQDs as inducers of a 2D structure and inhibitors of stacking to facilitate the formation of metal–organic framework (MOF) NSs [18]. This was achieved through a one-step room-temperature “bottom-up” synthesis strategy, eliminating the need for complex and low-yield exfoliation processes. GQDs possess a large specific surface energy and small size, which effectively prevent outgrowth agglomeration and promote the formation of NSs. Additionally, the introduction of carboxylated GQDs boosts the electrocatalytic performance of MOF NSs.

Inspired by previous work, herein, we report a facile *in-situ* GQDs-assisted hydrothermal method to synthesize near atom-layer 2H-MoS_2_ NSs (ALQD). A series of GQDs with different functional groups have been designed and fabricated [19–21]. Various techniques have been conducted to unravel the morphology, composition, and structure of as-prepared ALQD, including scanning transmission electron microscopy (TEM), atomic force microscope (AFM), X-ray photoelectron spectroscopy (XPS), Raman spectroscopy, and X-ray diffraction (XRD). The successful synthesis of the ALQD is attributed to the introduction of GQDs, which simultaneously enlarge the layer space and lower the formation energy of ALQD, guided by our theoretical calculations. As a proof-of-concept application, the hydrothermal-grown ALQD demonstrate superior HER performance compared to bulk MoS_2_ and pristine 2H-MoS_2_ NSs. Our study provides a landmark hint in the development of atom-layer semiconductor electrocatalysts for hydrogen production from water.

## Experimental Section

### Synthesis of SO_3_-GQDs

We obtained SO_3_-GQDs by a simple one-step method, based on the hydrothermal molecular fusion route. Yellow 1,3,6-trinitropyridine (0.5 g) and Na_2_SO_3_ (0.5 g) were dispersed in 50 mL deionized water and stirred for 0.5 h. The suspension was transferred to a 100-mL autoclave Teflon-lined stainless-steel autoclave and heated at 180 °C for 12 h. After cooling to room temperature, the clear solution obtained by filtration was dialyzed with a dialysis bag for three days to remove unreacted sodium salt, and dried at 60 °C. The synthesis process of other SO_3_-GQDs is similar to that of the process, except the added different sulfonic precursors: 4-hydrazinobenzenesulfonic acid for SO_3_-GQDs-1, 4-hydroxybenzenesulfonic acid for SO_3_-GQDs-2, and 1-amino-2-naphthol-4-sulfonic acid for SO_3_-GQDs-3.

### Synthesis of NH_2_-GQDs

Imitating the synthesis process of SO_3_-GQDs, we replaced the sulfonic acid source with equal mass NH_3_·H_2_O and got NH_2_-CQDs.

### Synthesis of OH-GQDs

Imitating the synthesis process of SO_3_-GQDs, we replaced the sulfonic acid source with NaOH, then the obtained powder named O-GQDs solved in the mixture of DMF and pure water in the ratio of five to one, and transferred into a 100-mL autoclave Teflon-lined stainless-steel autoclave and heated at 200 °C for 10 h. Finally, the OH-CQDs were gained.

### Synthesis of COOH-GQDs

The O-GQDs powder is completely dissolved in water, then sulfuric acid is dripped into the solution until pH = 2 ~ 3. The acidulated solution hydrothermal was transferred to a 100-mL autoclave Teflon-lined stainless-steel autoclave and heated at 180 °C for 10 h to obtain COOH-GQDs.

### Preparation of Bulk MoS_2_ and ALQD

Typically, 1.235 g (NH_4_)_6_Mo_7_O_24_·4H_2_O (1 mmol), 2.28 g thiourea (30 mmol) and 0.1 g SO_3_-GQDs powder is dissolved in 40 mL distilled water. The suspension was stirred for 1 h to form a homogeneous solution. The solution was transferred to a 100-mL Teflon-lined stainless-steel autoclave and heated at 200 °C for 20 h. After naturally cooled to room temperature, the product was collected by centrifugation and washed with ethanol. The purified black ALQD-SO_3_ was dried at 60 °C for structural characterization and property measurement. The other products (ALQD-SO_3_-1, ALQD-SO_3_-2, ALQD-SO_3_-3, ALQD-COOH, ALQD-OH, ALQD-NH_2_) are obtained by an analogical routine, replacing SO_3_-GQDs with corresponding GQDs. The synthesis process of bulk MoS_2_ is similar to that of the process, except the exclusion of SO_3_-GQDs.

### Electrochemical Measurements

All the electrochemical measurements were performed in a three-electrode system on an electrochemical work- station (CHI760e, Shanghai Chenhua Instruments Co., China) at room temperature. All tests above without iR-compensation. The electrocatalyst (5.0 mg) was dissolved in the mixture of 700 μL deionized water, 250 μL isopropyl alcohol, and 50 μL 5% Nafion solution, and then the electrocatalytic ink for all electrochemical tests was obtained. More importantly, the catalyst ink should be ultrasonically treated in an ice bath for 1 h to be fully mixed. Then, the aqueous dispersion of the catalyst (10 μL, 5.0 mg mL^−1^) was completely dropped to the glassy carbon electrode. We applied a rotating disk electrode (RDE) to proceed HER performance testing in a three-electrode cell configuration. A saturated Hg/Hg_2_Cl_2_ electrode (acid electrolyte) as the reference electrode, a graphite rod as the counter electrode, and the glassy carbon electrode supported catalysts as the working electrode. All potentials were calibrated to the RHE by the following equation:

1 $$ E_{{{\text{RHE}}}} = E\left( {{\text{Hg/Hg}}_{2} {\text{Cl}}_{2} } \right) + 0.059\,{\text{pH}} + 0.244 $$

The HER performance was measured in N_2_ saturated aqueous 0.5 M H_2_SO_4_ (pH = 0.3) at a scan rate of 50 mV s^−1^ with 100 cyclic voltammetry (CV) cycles in the range of 0.3 to − 0.8 V RHE. Linear sweep voltammetry (LSV) with a scan rate of 10 mV s^−1^ at 1600 rpm after 100 CV cycles in the range of 0.3 to − 0.8 V RHE. Tafel curves were then obtained from linear sweep voltammograms using a scan rate of 10 mV s^−1^. The electrochemical impedance spectroscopy (EIS) was executed in the frequency range of 100 kHz to 1 mHz with a modulation amplitude of 10 mV.

Cycling stability tests. The LSV curves were recorded for the first cycle and after 1000 CV sweeps between 0.3 and − 0.8 V RHE at 50 mV s^−1^. All the LSV curves were performed in the different electrolytes at a scan rate of 10 mV s^−1^.

Durability measurements. The chronoamperometry test of catalysts runs for no less than 20 h under the condition of constant overpotential at 245 mV, corresponding to current density of 10 mA cm^−2^ (η10).

### Material Characterization

Transmission electron microscopy (TEM) measurements were performed on a JEOL JEM-2100F electron microscope operating at 200 kV. The atomic force microscope (AFM) images were characterized using Bruker Dimension Icon AFM. The crystal phase structures of samples were investigated through X-ray diffraction, with a nickel-filtered Cu-Kα radiation source (XRD: Rigaku D/max-2500, Japan). The functional groups on the sample surface were measured by X-ray photoelectron spectroscopy (XPS, Thermo Fischer, ESCALAB250Xi). Raman spectra were assessed using Cora 5001 (Anton Paar OptoTec GmbH), which was implemented utilizing 785 nm as the excitation light source. Fourier transform infrared (FTIR) spectra were measuremed on a Nicolet iS50 spectrometer (Thermofisher) with a resolution of 0.09 cm^−1^ and a DLaTGS detector within the range 4000–400 cm^−1^. The temperature-programmed de-sorption (TPD) tests were conducted utilizing a Micromeritics Anton-Paar ChemBET characterization system with a quartz U-tube reactor and detected using a TCD. The electron conductivity of samples was characterized by a four-probe powder resistivity tester (Suzhou Jingge Electronic Co., Ltd. ST2722). The contact angle measurements are typically done by using Theta Flow equipment (Biolin Scientific) with optical and force tensiometers to perform calculations.

### Computational Details

The DFT calculations were implemented in the Vienna ab initio Simulation Package (VASP) [22]. The Perdew-Burke-Ernzerhof (PBE) method was adopted to establish the exchange correlation functional [23]. The energy cutoff was set to 500 eV and the Gaussian smearing was used (SIGMA = 0.05 eV). For all configuration, 2 × 3 × 4 Gamma K-point grid was constructed by vaspkit. In the calculation process, the dispersion interaction was corrected by DFT-D2 method [24]. The DFT calculations were considered to be convergent when the forces and the energy less than 0.02 eV Å^−1^ and 10^−5^ eV atom^−1^, respectively. For the Gibbs free energy calculations of HER process, the computational hydrogen electrode (CHE) method proposed by Nørskov was used [25]. The Gibbs free energy is given by Eq. (2):

2 $$ G = E + E_{{{\text{ZPE}}}} + \int {C_{{\text{p}}} {\text{d}}T} - TS $$

where *E* and *E*_ZPE_ are the total energy of system and the zero-point energy, respectively. *C*_p_ is the heat capacity; *T* is the temperature (298.15 K), and *S* is the entropy. The configuration of 2H-MoS_2_ was exported from Materials Project, and a 10 × 4 × 1 supercell was constructed in our DFT calculations. The GQDs consist of 102 C atoms, the edge is saturated by adding H atom. The size of GQDs was 1.36 × 2.17 nm. The functionalized GQDs were constructed by replacing one H atoms with the functional group. The small (0.51 × 0.94 nm) and medium sizes (0.94 × 1.93 nm) of GQDs consist of 84 and 27 C atoms, respectively. For the calculations of layer spacing, the lattice parameters were relaxed during the DFT calculations. For the Gibbs free energy calculations, the lattice parameters were fixed.

The MD simulations were implemented by Forcite module in Materials Studio. The universal forcefield was selected for the MD simulation. The Ewald method was used to handle the long-range electrostatic interactions with a cutoff radius of 1.2 nm [26]. The atom-based summation method was employed to treat the van der Waals interaction with a cutoff radius of 1.8 nm. The Nose thermostat was selected to control the temperature for dynamics simulation [27]. The time step is set as 2 fs for all the simulations. The structures of GQDs vertical/parallel insertion 2H-MoS_2_ were considered in 6.42 × 5.11 × 2.13 nm (vertical) and 6.42 × 5.11 × 0.99 nm (parallel). The total atom number of 1483 for two systems. The layer spacings of 2H-MoS_2_ were obtained by DFT calculations. All systems were first exposed to energy minimization by steepest descent method. Then, the systems were equilibrated for 5 ns in NPT ensemble. After that, production MD runs were performed for 15 ns in the NPT ensemble.

## Results and Discussion

### Theoretical Prediction of the Optimum Structure of ALQD-SO_3_

**Fig. 1:**
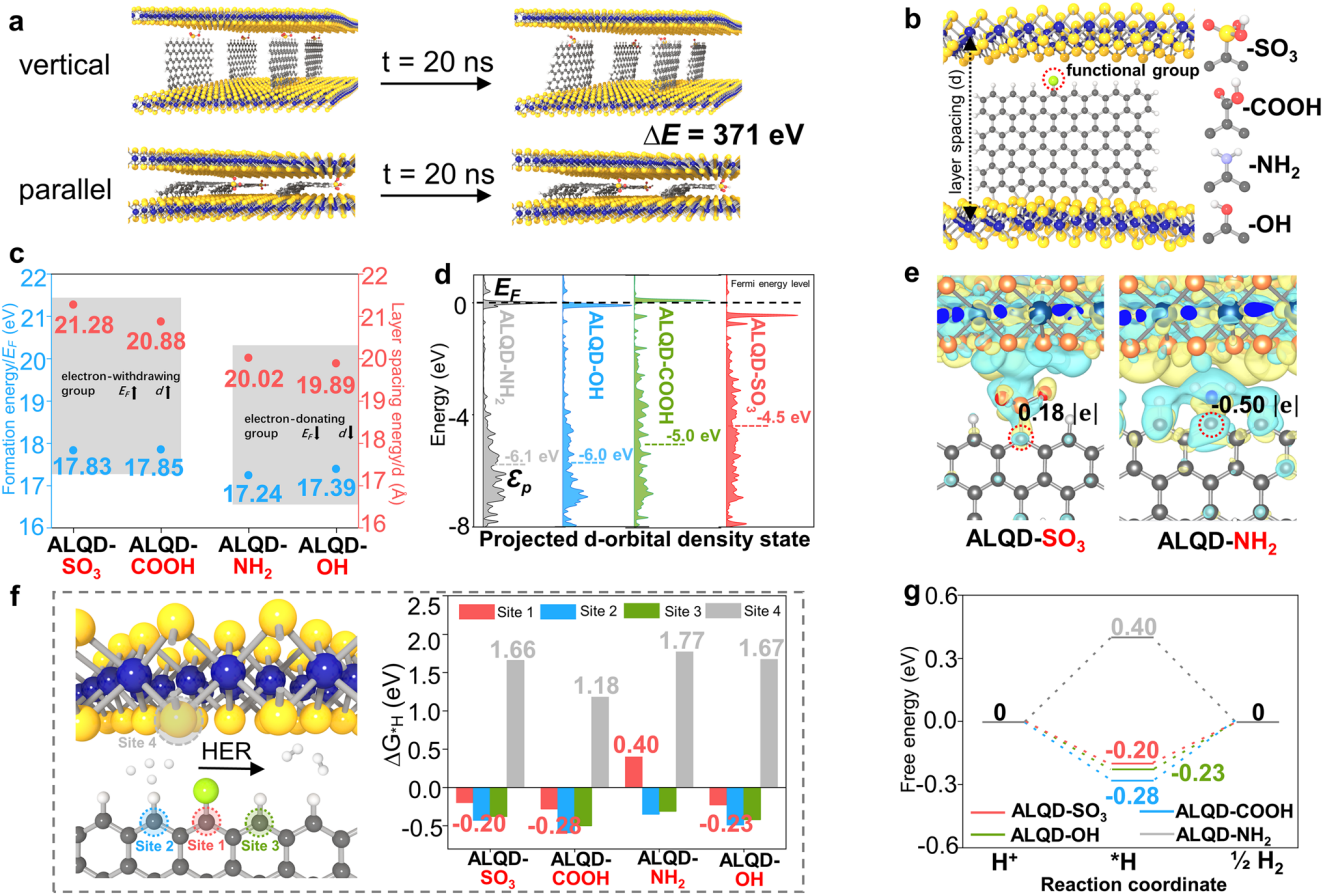
Structures, charge properties and HER performance of materials by theoretical prediction. **a** Structure stability of GQDs into the 2H-MoS_2_. **b** Structures of four types of ALQD, *d* is the layer spacing of 2H-MoS_2_ NSs by measuring the vertical distance of two Mo atoms. The yellow, blue, grey, white, and green ball represent S, Mo, C, H, and functional group, respectively. **c** Layer spacing and formation energy of four types of ALQD. **d** PDOS and p-band center of C atom connected to the -SO_3_ and -NH_2_. **e** Difference charge density and Bader charge of C atom connected to the -SO_3_ and -NH_2_. The green and red isosurface denote the decrease and increase in electron density, respectively, and the value is ± 3 × 10^−4^ e Bohr^−3^. **f** Gibbs free energy change (ΔG_*H_) of HER process on four sites of four types of ALQD. **g** Gibbs free energy profile of HER process for the C sites connected to the functional groups on four types of ALQD

The bulk MoS_2_ is generally obtained by the direct hydrothermal fabrication, which exhibits the inadequate HER performance. Building upon the success in preparing MOF NSs through the introduction of GQDs in the synthesis procedure, we employed theoretical calculations to predict the structures and formation energies of four types of ALQD. Furthermore, molecular dynamics (MD) simulation confirmed the structural stability of GQDs in 2H-MoS_2_. As schematically illustrated in Fig. 1a, the energy of GQDs with vertical insertion in 2H-MoS_2_ is lower than that of GQDs with parallel insertion (Δ*E* = 371 eV), indicating that the former is the more stable structure model. The MD results were used to establish the structures of ALQD for density functional theory (DFT) calculation, which are depicted in Fig. 1b.

The layer spacing of 2H-MoS_2_ upon introduction of GQDs was first investigated. As shown in Fig. 1c, the layer spacing (*d*) of 2H-MoS_2_ increases upon the introduction of electron-withdrawing group functionalized GQDs, which is larger than that of electron-donating group functionalized GQDs. Specifically, ALQD-SO_3_ exhibits the largest layer spacing of 21.28 Å, while ALQD-OH presents the smallest layer spacing of 19.89 Å. These findings agree well with prior research [18], which demonstrate that increased layer spacing can prevent NS stacking. The calculation results further reveal that the introduction of GQDs-SO_3_ in 2H-MoS_2_ can effectively inhibit stacking and promote the formation of 2D 2H-MoS_2_ NSs. Additionally, the formation energies of the four different types of ALQD were calculated, providing further evidence for the observed differences in layer spacing. Introducing electron-withdrawing group functionalized GQDs into 2H-MoS_2_ NSs requires more energy to expand the layer spacing compared to the introduction of electron-donating group functionalized GQDs, as evidenced by the higher formation energy of ALQD-SO_3_/COOH compared to ALQD-NH_2_/OH (0.44–0.65 eV).

The introduction of functionalized GQDs in 2H-MoS_2_ NSs affects not only the layer spacing, but also the electronic structure of ALQD, leading to improved electrocatalytic performance. The partial density of states (PDOS) and p-band center (ε_p_) of the C site directly linked to the functional group (C-SO_3_ and C-NH_2_) on GQDs were evaluated, as shown in Fig. 1d. The ε_p_ value of the active site close to the Fermi energy level is crucial for strong adsorption for intermediates [28, 29]. The results demonstrate that the ε_p_ value of C-SO_3_ (-4.5 eV) is closer to the Fermi energy level than that of C-NH_2_ (-6.1 eV). Notably, the ε_p_ value of C-COOH is 1.0 eV lower than that of C–OH (Fig. S1). The data of p-band center indicate that 2H-MoS_2_ NSs introduced by electron-withdrawing group functionalized GQDs possess enhanced adsorption capacity for intermediates and exhibit superior electrocatalytic performance. The Bader charge analysis also reveals strong electron transfer between the C atom and functional group (Fig. 1e). The C atom linked to the -SO_3_ functional group obtains 0.18 |e|, while the C-NH_2_ loses 0.50 |e|. Similar behavior is observed for C-COOH and C–OH (Fig. S1). Increased electrons at the active sites (C-SO_3_/COOH) can enhance the electrocatalytic performance, such as HER. In addition, the change of the charge distribution on the surface of 2H-MoS_2_ NSs through the functional group of GQDs are evident from the difference charge density, indicating that 2H-MoS_2_ NSs can be activated by electron-withdrawing groups on GQDs.

Considering the electrocatalytic performance of materials is strongly affected by their structure and electronic properties, the Gibbs free energy change (ΔG_*H_) of the HER process was calculated for four kinds of ALQD in Figs. 1f and S2-S5. Four different sites on the ALQD were considered: C site connected to the functional group (site 1), two C sites near the functional group (site 2, 3) and S site (site 4). The C sites connected to the functional group are found to be the active sites for the HER process, except for ALQD-NH_2_ (Fig. 1f). The Bader charges of C atoms near -NH_2_ (0.08 |e| and -0.01 |e|) are more positive than those of C atoms connected to -NH_2_ (-0.50 |e|), suggesting that C atoms near -NH_2_ are the active sites for the HER process (Fig. S6). In addition, the S site of the ALQD-COOH exhibits superior HER performance compared to other ALQD, indicating that the electron-withdrawing -COOH group can activate the S sites of 2H-MoS_2_.

Figure 1g presents the Gibbs free energy profile of the HER process for the C sites connected to the functional group. ALQD-SO_3_ exhibits superior HER performance because of its lowest ΔG_*H_ value of -0.20 eV, while ALQD-NH_2_ shows poor HER performance. Interestingly, ALQD-OH performs better in the HER process compared to ALQD-COOH, despite the relatively small differences in their ΔG_*H_ values (0.03–0.08 eV). This should be attributed to the potential impact of the size of GQDs on the regulation effect of functional groups. To evidence this hypothesis, the ΔG_*H_ values for different-sized ALQD (small size: 0.51 × 0.94 nm; medium size: 0.94 × 1.93 nm) were calculated (Figs. S7 and S8). The trend is observed for the medium-sized ALQD: ΔG_*H_ increases from electron-withdrawing group functionalized ALQD to electron-donating group functionalized ALQD. However, there is a significant difference in the ΔG_*H_ values of the four types of small-sized ALQD. Specifically, ALQD-SO_3_ and ALQD-NH_2_ show the minimum (-0.18 eV) and maximum (0.48 eV) ΔG_*H_ values, respectively. Based on these results, ALQD-SO_3_ should be the most promising one to display optimal HER performance among the four types of ALQD.

### Morphological and Structural Characterization of ALQD-SO_3_

**Fig. 2:**
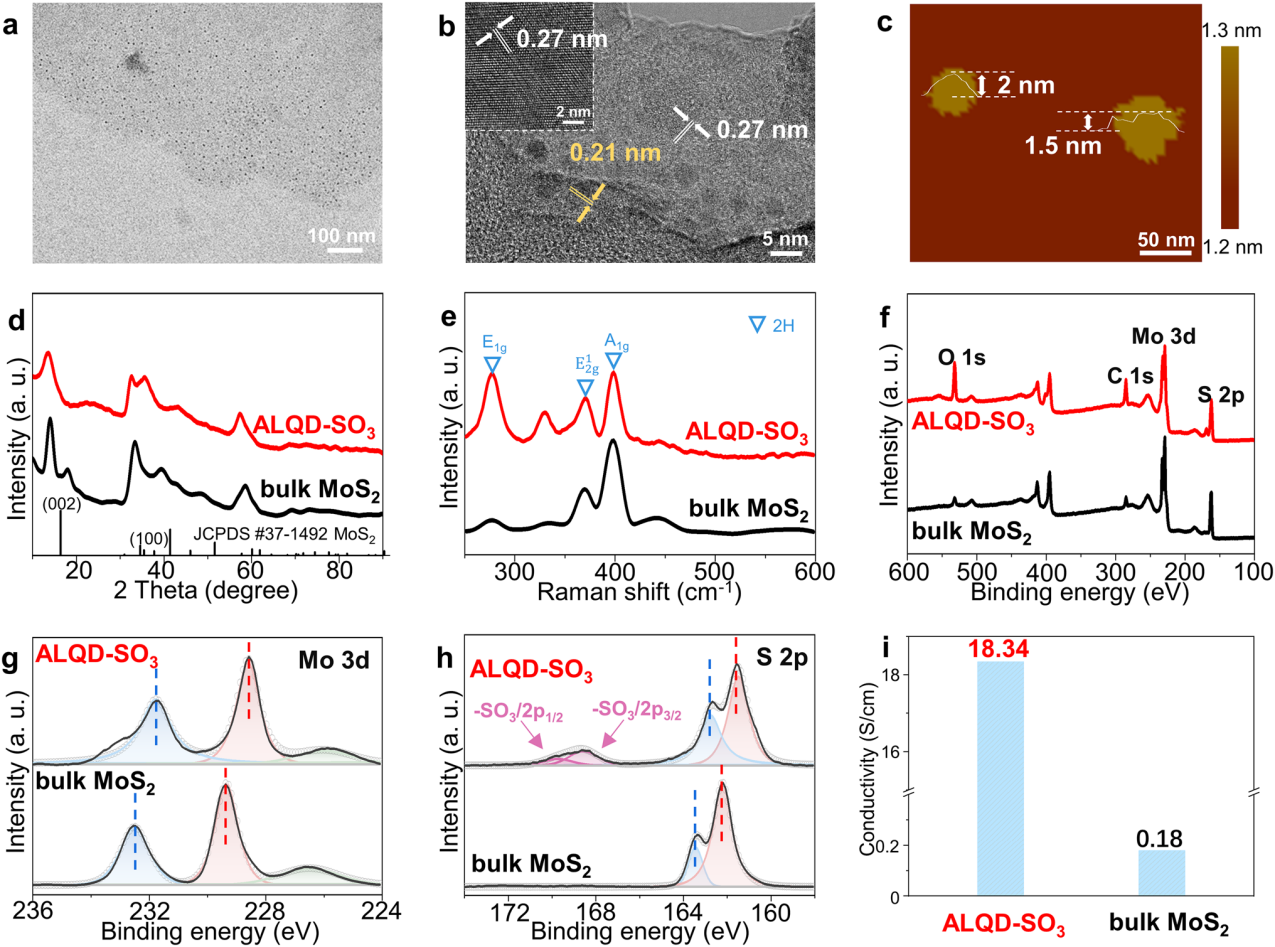
Morphological and structural characterization of synthesized materials. **a** TEM image of ALQD-SO_3_. **b** High-resolution TEM image of ALQD-SO_3_. The inset in **b** shows the regular lattice of zoomed-in MoS_2_ NSs. **c** AFM image of ALQD-SO_3_. **d** XRD patterns of ALQD-SO_3_ and bulk MoS_2_. **e** Raman spectra of ALQD-SO_3_ and bulk MoS_2_. **f** XPS survey, and high-resolution XPS **g** Mo 3*d* and **h** S 2*p* spectra of ALQD-SO_3_ and bulk MoS_2_. **i** Electrical conductivities of ALQD-SO_3_ and bulk MoS_2_

As schematically shown in Fig. 2, our *in-situ* synthesis strategy is based on a one-pot hydrothermal method that utilizes SO_3_-GQDs in the synthesis process to tune the thickness of the 2H-MoS_2_. We have achieved possible industrial preparation and obtained a large number of samples by one-time preparation, producing approximately about 1.3 g, with a production yield of 35.96% (Fig. S9). The unbound SO_3_-GQDs on the sample surface can be simply removed by the centrifugal cleaning by DI water, owing to the super-hydrophilic characteristics of SO_3_-GQDs [19]. A series of morphological and structural characterizations have been performed to investigate the 2D structure formation and conversion of ALQD-SO_3_. The TEM image of ALQD-SO_3_ shows a sheet structure (Fig. 2a), with SO_3_-GQDs uniformly attached to the surface of the ultrathin 2H-MoS_2_ NSs. The AFM image shown in Fig. 2c further reveals its uniform thickness with an average height of about 2 nm, further supporting the NS architecture of the as-prepared ALQD-SO_3_. Conversely, the bulk MoS_2_ exhibits an aggregation appearance, with a height as high as 120 nm, far away from the NS morphology (Figs. S10 and S11). The high-resolution TEM image (Fig. 2b) shows a lattice fringe of 0.27 nm attributed to the (100) plane of 2H-MoS_2_ [30]. Apart from the observed lattice of the primary NSs, a distinct lattice fringe of the introduced SO_3_-GQDs (0.21 nm) was found [31], which indicates that SO_3_-GQDs were successfully coupled to the 2H-MoS_2_ substrates. The high quality of the as-synthesized samples demonstrates the superiority of the GQD-induced facile hydrothermal synthesis method [12].

As shown in the XRD pattern (Fig. 2d), the peak position of bulk MoS_2_ prepared by our one-pot hydrothermal method without SO_3_-GQDs perfectly matches the standard pattern of 2H-MoS_2_. The strongest peak is noticeable around 14° wide (002) peak, which is attributed to the interplanar spacing (6.15 Å) [32]. The interplanar spacing (2.7 Å) of the (100) peak located at 2θ≈32° [33], is consistent with high-resolution TEM image. However, the (002) peak intensity of as-prepared samples through SO_3_-GQDs regulation is dramatically weakened, demonstrating a few-layer graphene-like stacked structure [34]. Furthermore, the strongest diffraction peak of ALQD-SO_3_ exhibits a blue-shift compared to that of bulk MoS_2_, suggesting an increased interlayer distance in ALQD-SO_3_ [35]. This serves as crucial evidence for the formation of NS architectures caused by the opening interlayer spacing of SO_3_-GQDs. The structures of bulk MoS_2_ and ALQD-SO_3_ are further analyzed by their Raman spectra (Fig. 2e), which exhibit two characteristic peaks of 2H-MoS_2_ at 376 and 398 cm^−1^, originating from the in-plane stretching and out-of-plane vibration Raman modes of S–Mo–S, respectively [36, 37]. The extra peaks demonstrate that the presence of GQDs in ALQD induced structural configuration changes leads to a distorted 2H phase in the basal plane of MoS_2_, thereby revealing the coordination of MoS_2_ with GQDs on the ALQD nanosheets [38].

The chemical nature and stoichiometric compositions of as-prepared samples via SO_3_-GQDs-regulation were confirmed by XPS (Fig. 2f–h). The deconvoluted S 2*p* spectrum of ALQD-SO_3_ contains the peak at ≈168 eV corresponding to SO_3_ configurations, implying the presence of SO_3_-GQDs [18]. The deconvoluted Mo 3*d* peaks possess two peaks at 229.4 and 232.2 eV, corresponding to Mo^4+^ 3*d*_5/2_ and 3*d*_3/2_ of the 2H phase, respectively, confirming the 2H phase of bulk MoS_2_ [39, 40]. Additionally, a blueshift of ≈0.8 eV to lower binding energy (228.6 and 231.8 eV) is observed in ALQD-SO_3_. This shift phenomenon can be ascribed to the introduction of electron-withdrawing group functionalized SO_3_-GQDs, leading to the increase in the electron density around the Mo and S sites, consistent with the result of our theoretical prediction (Fig. S3) [41, 42]. According to the stoichiometry analysis result (Table S1), the Mo:S molar ratio decreases significantly from about 1:1.59 to 1:1.35 after SO_3_-GQDs-regulation. The storage stability of the synthesized samples was further investigated by XPS measurement. As shown in Fig. S12, the spectra of ALQD-SO_3_ produced 6 months ago and freshly produced exhibit no significant difference, demonstrating the excellent storage stability of our designed materials.

Notably, the electrical conductivity of ALQD-SO_3_ is two orders of magnitude higher than that of bulk MoS_2_ (18.34 vs 0.18 S cm^−1^), which is beneficial for the charge transfer kinetics in HER process (Fig. 2i) [43]. In electrocatalyst applications, the hydrophilic property increases the interfacial contact area between the catalyst surface and aqueous electrolyte, thereby improving the overall catalytic performance [44]. Unignored, the higher hydrophilicity of ALQD-SO_3_ than that of bulk MoS_2_ further verifies the introduction of the electron-withdrawing group functionalized SO_3_-GQDs can enhance the electrocatalysis activities of the designed ALQD-SO_3_ (Fig. S13). In light of these analyses, the results corroborate the more efficient HER application of semiconducting ALQD-SO_3_ [45, 46].

### Evaluation Toward Electrochemical Hydrogen Evolution

**Fig. 3:**
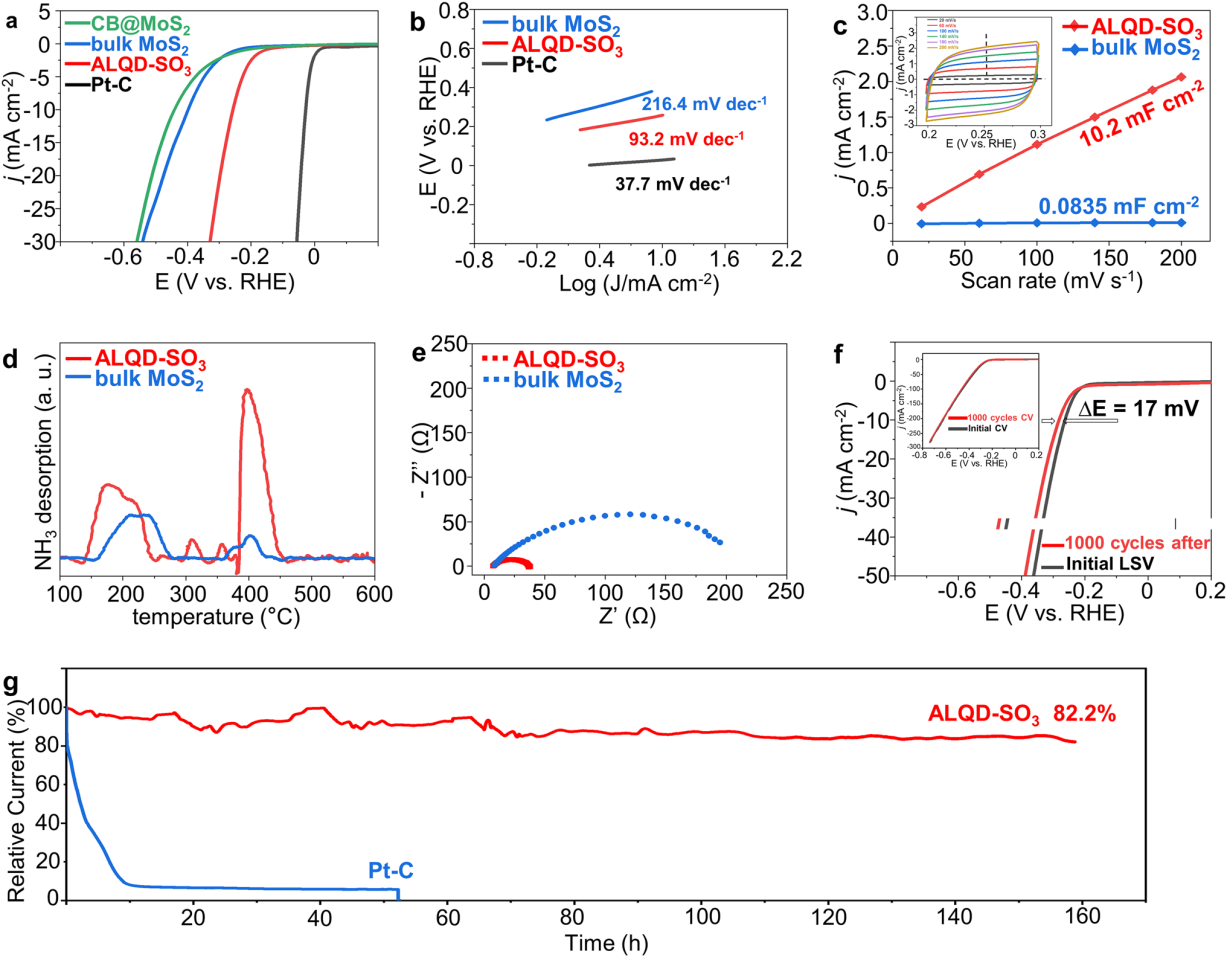
HER performance of synthesized materials. **a** LSV curves of CB@MoS_2_, bulk MoS_2_, ALQD-SO_3_ and PtC were performed in 0.5 M H_2_SO_4_ electrolyte. **b** The corresponding Tafel slopes were derived from LSV curves. **c** The ratio of the capacitive currents measured at 0.25 V vs RHE for bulk MoS_2_ and ALQD-SO_3_.The inset in **c** shows cyclic voltammograms of ALQD-SO_3_ at a scan rate from 20 to 200 mV s^−1^. **d** NH_3_-TPD profiles of bulk MoS_2_ and ALQD-SO_3_. **e** Nyquist plots of bulk MoS_2_ and ALQD-SO_3_. **f** LSV curves measured before and after 1000 cycles at 0.2 to -0.8 V versus RHE. **g** Time dependence of current density at 250 mV versus RHE

To evaluate the influence of SO_3_-GQDs on HER performance, the electrochemical characteristics of various MoS_2_ samples were measured using a rotating disk electrode (RDE) at a rate of 1600 rpm in 0.5 M H_2_SO_4_ (see the Materials and Methods section for details). Linear sweep voltammetry (LSV) curves are exhibited in Fig. 3a. The overpotentials (η) at a current density of 10 mA cm^−2^ of ALQD-SO_3_ NSs is 1.85 times lower than bulk MoS_2_ (245 and 453 mV vs RHE), which is consistent with the theoretical calculations of ΔG_*H_ = -0.20 eV. Moreover, we synthesized a sample using the equal mass carbon black (CB) instead of SO_3_-GQDs in the material synthesis procedure, and the as-synthesized sample exhibited poor HER activity similar to that of bulk MoS_2_, implying that the regulation of the SO_3_-functional group of GQDs plays the pivotal role in facilitating the HER capacities of MoS_2_. To investigate the kinetic metrics, the Tafel slope is used to determine the rate-determining step for HER. From the extrapolation of the linear region of overpotential (η) versus log*j* (Fig. 3b), we obtained Tafel slopes of 93.2 and 216.4 mV per decade for ALQD-SO_3_ and bulk MoS_2_, respectively. These results suggest that the role of GQDs in regulating MoS_2_ electron is essential for improving the catalytic performance of HER. Both theoretical and experimental findings support the conclusion that ALQD-SO_3_ exhibit superior HER catalytic activity and more effective active sites due to their unique structural characteristics.

To evaluate the effective electrochemical active surface areas (ECSAs) of the as-prepared electrocatalytic materials, cyclic voltammograms (CV) at different scan rates, and double-layer capacitances (C_dl_) were measure. Figure 3c illustrates that the highest value of ECSA is observed for ALQD-SO_3_, which correlated with its superior HER performance. The ECSA value of ALQD-SO_3_ (10.2 mF cm^−2^) is about three orders of magnitude higher than that of bulk MoS_2_ (0.0835 mF cm^−2^), indicating that the introduction of SO_3_-GQDs in MoS_2_ can significantly increase the ECSAs and expose more active sites for the electrochemical catalytic reaction. TPD measurements using NH_3_ as a probe molecule were performed to further determine the type of active sites of the catalyst [38]. Figure 3d shows that bulk MoS_2_ exhibits a weak Lewis acidic site to the NH_3_ adsorption sites at 230 and 400 °C. However, the ALQD-SO_3_ exhibits strong Lewis acidic sites to the NH_3_ adsorption site at the above temperature due to its distinctive structure. Furthermore, there are two novel NH_3_ adsorption sites at 310 and 360 °C owing to the addition of SO_3_-GQDs. It is widely accepted that high-acidity sites such as -SO_3_H groups of GQDs are the key to promoting HER activity. The Nyquist diagram in Fig. 3e shows that the charge transfer resistance (R_ct_) of 2H MoS_2_ (230 ω) is six times larger than that of ALQD-SO_3_ (36 ω), indicating that appropriate SO_3_-GQDs can accelerate interfacial electron transfer kinetics and charge transfer capacity to optimize HER activity [47, 48]. These results demonstrate the significantly improved intrinsic properties of ALQD-SO_3_, providing a favorable platform for electrocatalytic activities.

In addition to the remarkable improvement of HER performance, we also evaluated their long-term cycling stability by conducting the CV process for 1000 cycles [49]. As depicted in Fig. 3f, the LSV curve of ALQD-SO_3_ after 1000 cycles is very close to the initial one, demonstrating its remarkable stability as an HER electrocatalyst. The inset shows the CV profiles of the first and 1000th cycles, revealing no visible degradation. Moreover, the catalytic stability of ALQD-SO_3_ was studied by chronoamperometry (*i–t*), where the voltage was fixed at 0.25 V (vs RHE). Compared with Pt/C, ALQD-SO_3_ exhibit greater durability and a higher current retention rate (82.2%) after 160 h of continuous operation (Fig. 3g). Such outstanding long-term catalytic stability suggests that our ALQD-SO_3_ can serve as a high-efficiency HER electrocatalyst for large-scale practical applications.

To further confirm that the enhanced HER activity originates from the –SO_3_ functionality, we designed and synthesized a series of SO_3_-GQDs with different precursors (named SO_3_-GQDs-x, *x* = 1,2,3) using the hydrothermal treatment similar to the preparation of aforementioned SO_3_-GQDs. TEM results show that all SO_3_-GQDs-x samples have a similar lateral size distribution to SO_3_-GQDs, as well as an intact graphene crystal structure (Fig. S14). XRD analysis reveals a broad (002) peak at around 2*θ* = 25.4° for all SO_3_-GQDs-x samples (Fig. S15). As revealed by FTIR spectra, in addition to the -SO_3_H and -OH bonds at round 900–1200 and 3300–3500 cm^−1^, the surface of the newly synthesized three SO_3_-GQDs-x samples also possess NH_2_ (≈3180 cm^−1^) functional groups (Fig. S16) originating from their precursors, which is further confirmed by XPS measurements (Fig. S17) [50, 51]. According to the deconvolution results of XPS S 2p spectra, the -SO_3_H content increases in the order of SO_3_-GQDs-3 (2.15 at%) < SO_3_-GQDs-2 (4.10 at%) < SO_3_-GQDs-1 (4.39 at%) < SO_3_-GQDs (7.49 at%) (Fig. S17 and Table S2). To modulate the electronic structure of MoS_2_, these SO_3_-GQDs-x were introduced into the preparation of MoS_2_ as a robust regulator (denoted as ALQD-SO_3_-1, ALQD-SO_3_-2, and ALQD-SO_3_-3). TEM images show that all the ALQD-SO_3_-x have a sheet morphology, similar to the basal morphology of ALQD-SO_3_ (Fig. S18), while their heights are thickened with decreasing content of -SO_3_H functional groups, as determined by their AFM images and XPS data (Figs. S19 and S20), demonstrating the thickness engineering of the functional GQDs to 2D nanomaterials. The phase structures of these ALQD-SO_3_-x do not undergo any transition upon the addition of these GQDs-SO_3_-x (Figs. S21 and S22), whereas the content of electro-withdrawing -SO_3_H group of ALQD-SO_3_-x follows the trend of SO_3_-GQDs-x (Fig. S20). Notably, the contact angle test also indicates that the hydrophobicity of ALQD-SO_3_-x strengthen with their decreased -SO_3_H group concentrations (Fig. S23). Furthermore, their overpotential at the current density of η10 emerged in a downward trend, which follows the same trend as the -SO_3_H content (Figs. S19 and S24). Based on the performance trend of the class of ALQD-SO_3_-x, we conclude that the electro-withdrawing -SO_3_ functionalized GQDs engineering strategy applies to MoS_2_ NS catalysts. The stronger the electron-withdrawing capability of the GQDs modulated MoS_2_ is, the more the HER performance is promoted [52].

### Classification of the Functional Groups of GQDs on 2H-MoS_2_

To further verify the effectiveness of electron-withdrawing group functionalized GQDs on MoS_2_ for HER applications, we synthesized electron-withdrawing -COOH group functionalized GQDs (COOH-GQDs) and electron-donating -OH group functionalized GQDs (OH-GQDs) using a redox method described in our previously reported work [20, 53]. The OH-GQDs and COOH-GQDs manifest a similar lateral size distribution to the aforementioned GQDs (Fig. S25), as well as the intact graphene crystal structure (Fig. S26). XPS analysis confirms the difference in functional groups between the two GQDs (Fig. S27 and Table S3), with the COOH-GQDs exhibiting a significantly higher content of C = O than C-O, while the opposite is true for the OH-GQDs, consistent with the chemical bonding characteristics of -COOH and -OH [54, 55].

**Fig. 4:**
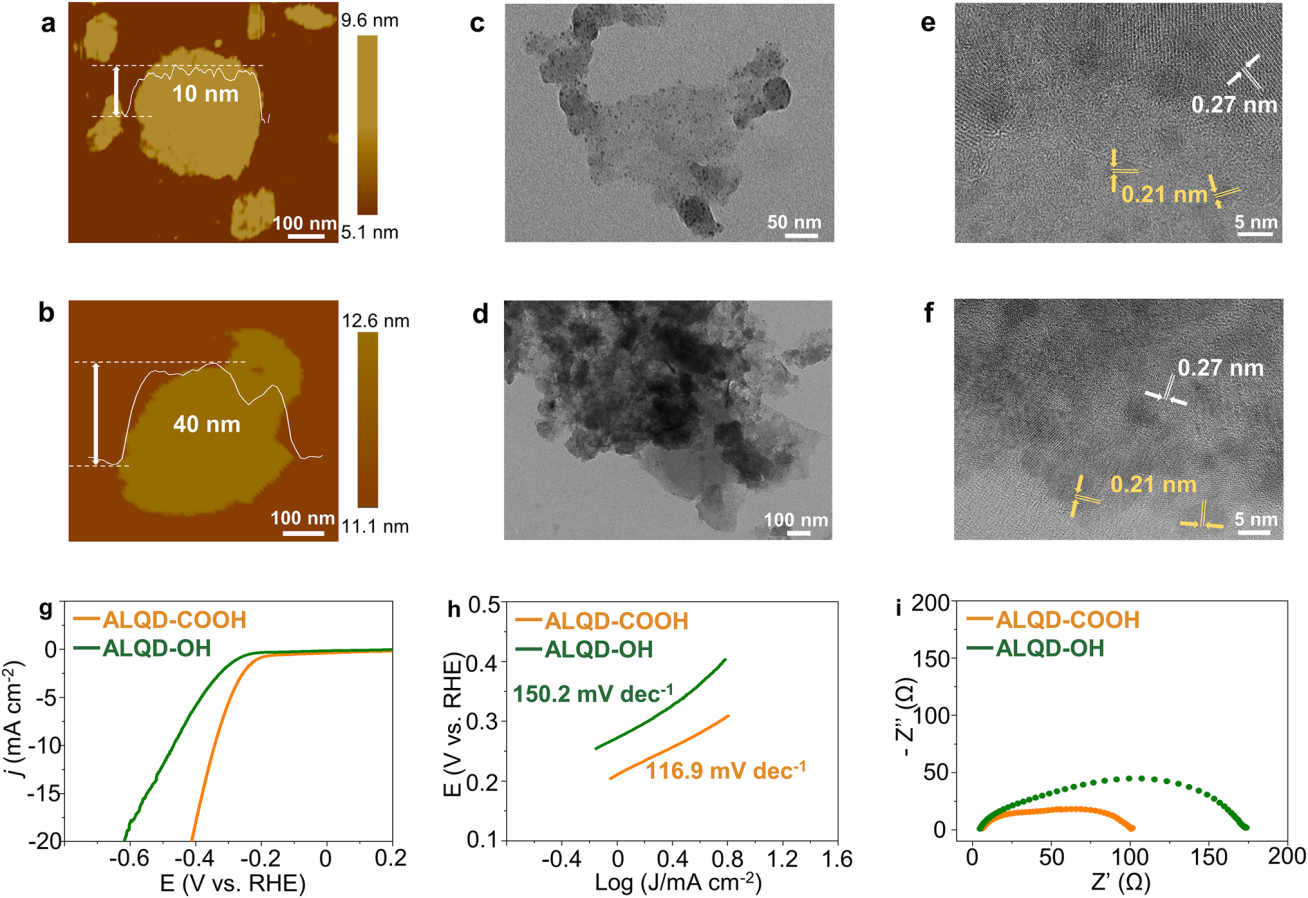
Morphological characterization and HER performance of ALQD-COOH and ALQD-OH. AFM images of** a** ALQD-COOH and **b** ALQD-OH. TEM images of **c** ALQD-COOH and **d** ALQD-OH. High-resolution TEM images of **e** ALQD-COOH and **f** ALQD-OH. **g** LSV curves of ALQD-COOH and ALQD-OH were performed in 0.5 M H_2_SO_4_ electrolyte. **h** The corresponding Tafel slopes were derived from LSV curves. **i** Nyquist plots of ALQD-COOH and ALQD-OH

Structural characterization and property measurement have been performed to study the corresponding products, i.e., ALQD-COOH and ALQD-OH. The thicknesses of the as-synthesized ALQD-COOH and ALQD-OH are about 10 and 40 nm, respectively (Fig. 4a, b), which agrees with the above-mentioned finding that the electron-withdrawing group functionalized GQDs induce the *in-situ* growth of thinner MoS_2_ NSs. TEM images (Fig. 4c, d) further confirm their sheet structure characteristics, with the ALQD-COOH displaying fewer-layer NSs and observable COOH-GQDs on its surface [56], while the ALQD-OH shows severe aggregation of NSs, consistent with their AFM data. High resolution TEM images (Fig. 4e, f) further reveal the presence of GQDs on NSs, and XRD pattern (Fig. S28) indicates that the peaks of ALQD-OH roughly match the standard pattern of 2H-MoS_2_. The strongest diffraction peak of ALQD-COOH is shifted to a lower degree than that of ALQD-OH, in accordance with the trend observed in ALQD-SO_3_. The Raman pattern (Fig. S29) clearly shows characteristic peaks at 376 and 398 cm^−1^ of ALQD-OH and ALQD-COOH, which sustains the 2H phase of 2H-MoS_2_ after the introduction of the functionalized GQDs. The chemical states of as-synthesized materials were identified by XPS (Fig. S30). The binding energy of Mo and S in ALQD-COOH is approximately 0.66 and 0.68 eV lower than that of ALQD-OH, indicating that GQDs-COOH can enhance the electron cloud density around the Mo and S atoms and boost their activities. Importantly, the ALQD-COOH introduced by electron-withdrawing -COOH group functionalized GQDs exhibits favorable hydrophilic properties (Fig. S31), and improves HER electrocatalytic performance.

In addition, we measured and analyzed the HER performance of the two samples. As demonstrated in Fig. 4g–i, the LSV curve illustrate the different in their HER performance, with overpotential at η10 being 345 and 468 mV for ALQD-COOH and ALQD-OH, respectively. It is worth noting that the HER activities of ALQD-COOH still exhibit a certain gap with ALQD-SO_3_ because of the weaker electron-withdrawing capability of the -COOH group than the –SO_3_ group. Tafel slope is extracted to investigate the reaction kinetics during HER (Fig. 4h) [57]. The Tafel slope of ALQD-COOH (116.9 mV dec^−1^) is smaller than that of ALQD-OH (150.2 mV dec^−1^), indicating that the Volmer-Tafel mechanism is the HER pathway. Furthermore, the better HER performance of ALQD-COOH also originates from the fast charge transfer and transport process, as evidenced by the electrochemical impedance spectra (Fig. 4i). Specifically, ALQD-COOH exhibits the lowest intrinsic resistance, as indicated by the steepest slope in the low-frequency region and the smallest charge transfer resistance indicated by the smallest semicircle in the high-frequency region [58]. All the above results together demonstrate that GQDs functionalized with electron-withdrawing groups such as -SO_3_ and -COOH can regulate the interlayer structure of MoS_2_ during the *in-situ* synthesis process, resulting in rapid charge transfer and superior HER capacities.

Another control experiment was carried out using the electron-donating NH_2_-functionalized GQDs (denoted as NH_2_-GQDs) to modify the properties of MoS_2_ [59, 60]. The NH_2_-GQDs was synthesized by introducing ammonia during the synthesis of SO_3_-GQDs (see Methods section for more details). The XPS results confirm that the –NH_2_ group is successfully decorated onto NH_2_-GQDs (4.19 at%) and ALQD-NH_2_ (0.66 at%) (Fig. S32). NH_2_-GQDs has the same graphene structure (Fig. S33) and comparable lateral size distribution as all the GQDs mentioned above (Fig. S34). The thickness of the fabricated ALQD-NH_2_ were measured about 65 nm (Fig. S35), which is high away that of ALQD-SO_3_. Similar to the role of electron-donating -OH group on the MoS_2_, ALQD-NH_2_ does not show any improvement in HER performance (Fig. S36). Based on the above characterization results, it can be concluded that incorporating GQDs functionalized with electron-donating group into MoS_2_ is detrimental to the HER performance of MoS_2_@GQDs catalysts.

## Conclusions

In summary, we firstly used theoretical calculation to predict the possible formation mechanism of 2H-MoS_2_ NSs induced by GQDs and the intrinsic mechanism for improving their HER performance. Our findings indicate that electron-withdrawing groups in GQDs can enlarge the layer spacing of 2H-MoS_2_, effectively stimulating the formation of the 2D 2H-MoS_2_ NSs. Additionally, we identified both Mo and S as potential active sites, activated by the electron-withdrawing groups of ALQD, which maintain a higher charge density of C atoms connected to the functional group, thereby leading to enhanced HER performance. Guided by our theoretical predictions, we successfully synthesized near atom-layer ALQD-SO_3_ using a functionalized GQD-induced *in-situ* bottom-up approach. The obtained ALQD-SO_3_ exhibited near atom-layer thickness about 2 nm and long-time storage stability. Remarkably, the synthesized near atom-layer ALQD-SO_3_ demonstrated significantly improved HER performance, delivering a low overpotential of 245 mV to reach a current density of 10 mA cm^−2^ and a small Tafel slope of 93.2 mV dec^−1^ with fast reaction kinetics. Furthermore, the superb electrocatalytic stability was achieved at least 160 h without sharp activity decay, which suggested its prospect applications as a high-performance electrocatalyst. We also unraveled the distinct effects of electron-withdrawing group functionalized GQDs and electron-donating group functionalized GQDs on the construction and HER activity of MoS_2_ NSs. Specifically, electron-withdrawing group functionalized GQDs facilitate the formation of NS architectures of MoS_2_ and HER proceeding, while electron-donating group functionalized GQDs favor the construction of aggregated bulk MoS_2_ and lead to sluggish HER kinetics.

Our results suggest that electron-withdrawing group functionalized GQDs play a vital role in regulating the electronic structure and morphology of 2H-MoS_2_. It is worth noting that the strength of the electron-withdrawing group on GQDs (-SO_3_ vs -COOH) affects the thickness and HER catalytic activity of the obtained 2H-MoS_2_ NSs. Therefore, it is worthwhile to explore the possible synthesis pathway of stronger electron-withdrawing groups (-CF_3_, -CN, etc.) functionalized GQDs for fabricating atom-layer 2H-MoS_2_ NSs with more robust electrocatalytic capacities in further study. Our approach enlightens the development of related atom-layer semiconductor electrocatalysts, making them easily accessible for further exploration of their exciting physicochemical properties and applications in electrochemical devices and catalysis. Furthermore, our approach can obtain a large amount of near atom-layer semiconductor electrocatalyst nanomaterials with a simple and safe hydrothermal operation in a short time, rendering it suitable for large-scale production with excellent industrialization prospects.

### Supplementary Information

Below is the link to the electronic supplementary material.

Supplementary file1 (Docx 12.2 MB)
