# Supplementary material for: Graphene Quantum Dot-Mediated Atom-Layer Semiconductor Electrocatalyst for Hydrogen Evolution
Source: Nanomicro Lett. 2023 Sep 28;15:217. doi: 10.1007/s40820-023-01182-7 (PMC10539274; doi:10.1007/s40820-023-01182-7)
Supplement: Supplementary file 1 — Supplementary file1 (Docx 12.2 MB) [file 40820_2023_1182_MOESM1_ESM.docx]

**Supplementary Information**

Graphene Quantum Dot Mediated Atom-Layer Semiconductor Electrocatalyst for Hydrogen Evolution

Bingjie Hu^1†^, Kai Huang^2†^, Bijun Tang^3^, Zhendong Lei^3^*, Zeming Wang^1^, Huazhang Guo^1^, Cheng Lian^2^*, Zheng Liu^3^*, Liang Wang^1,3^*

^1^Institute of Nanochemistry and Nanobiology, School of Environmental and Chemical Engineering, Shanghai University, 99 Shangda Road, BaoShan District, Shanghai 200444, P. R. China

^2^State Key Laboratory of Chemical Engineering, Shanghai Engineering Research Center of Hierarchical Nanomaterials, and School of Chemistry and Molecular Engineering, East China University of Science and Technology, Shanghai 200237, P. R. China

^3^School of Materials Science and Engineering, Nanyang Technological University, 50 Nanyang Avenue, Singapore 639798, Singapore

*Correspondence to: zhendong.lei@ntu.edu.sg (Z. Lei); liancheng@ecust.edu.cn (C. Lian); z.Liu@ntu.edu.sg (Z. Liu); wangl@shu.edu.cn (L. Wang).

†These authors contributed equally to this work

**This PDF file includes:**

Supplementary Figures. 1-36

Supplementary Tables 1-3


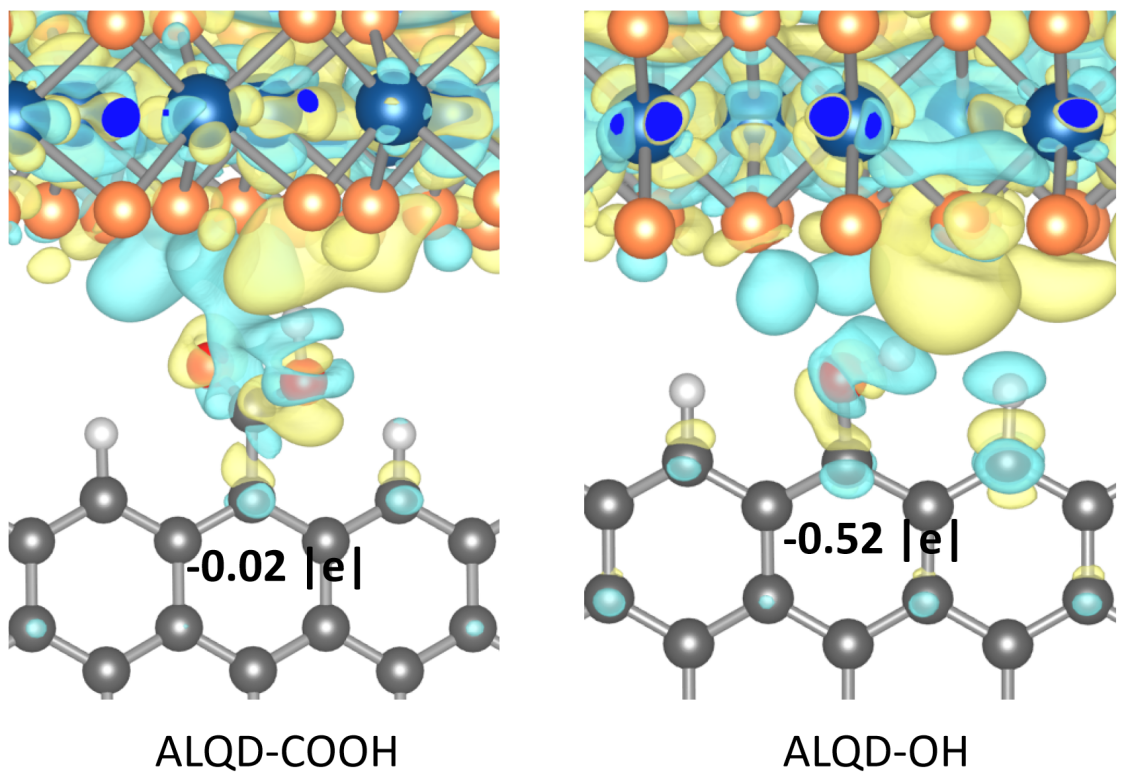


**Fig. S1** Difference charge density and bader charge of C atom connected to the -COOH and -OH. The green and red isosurface denote the decrease and increase of electron density, respectively, and the value is ±3 × 10^-4^ e Bohr^-3^.


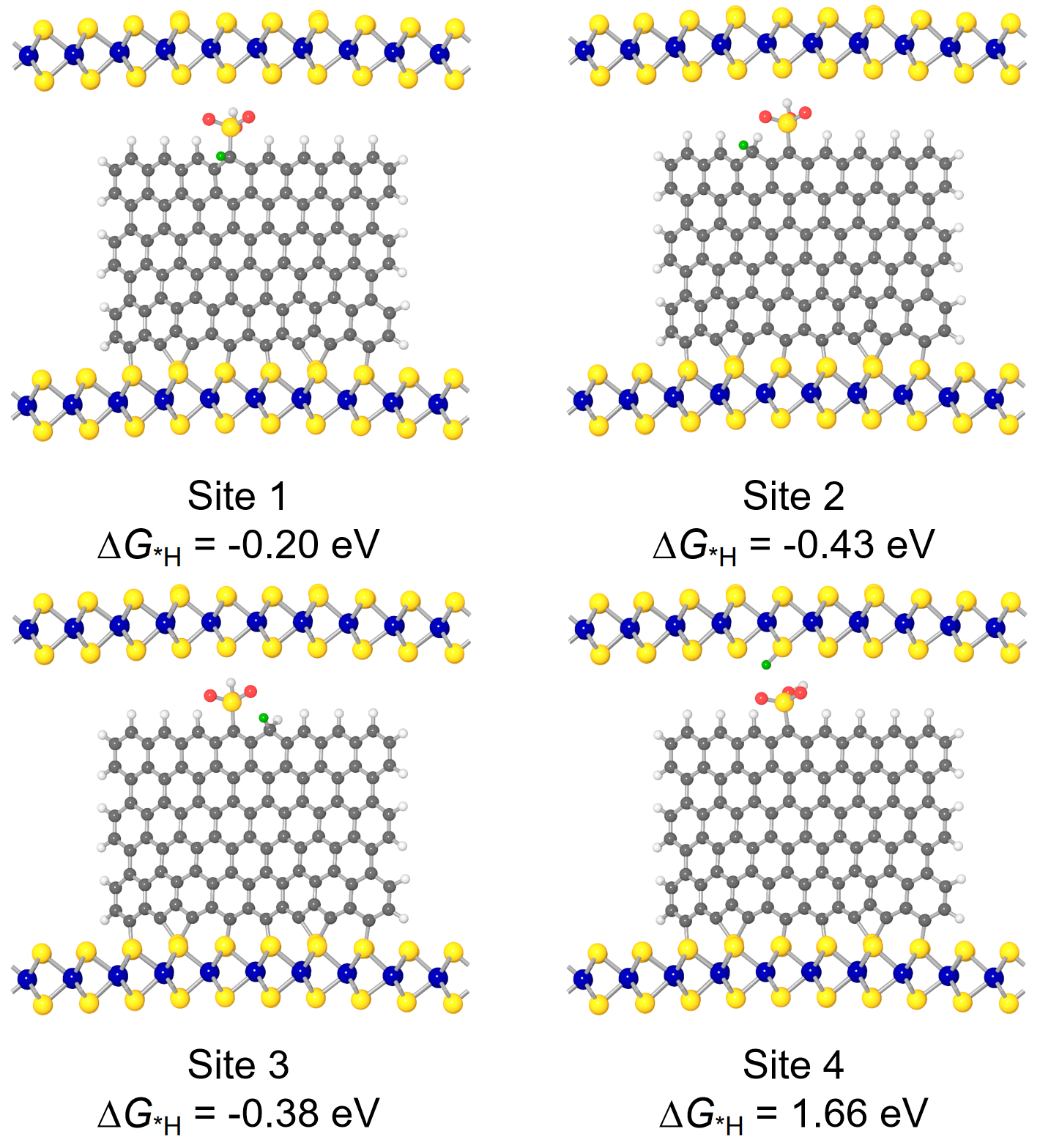


**Fig. S2** Gibbs free energy change (Δ*G*_*H_) of HER process on four sites of ALQD-SO_3_.


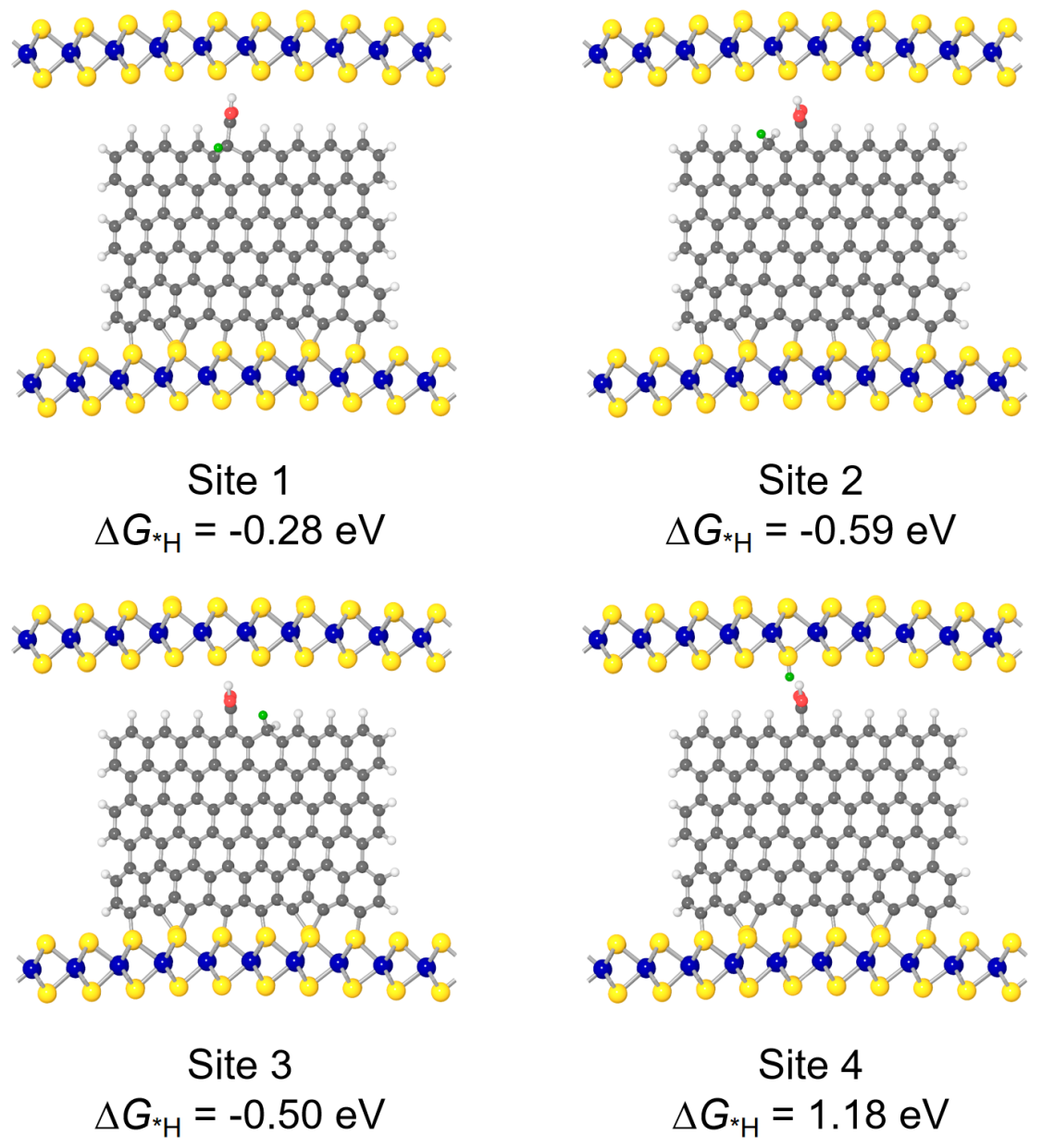


**Fig. S3** Gibbs free energy change (Δ*G*_*H_) of HER process on four sites of ALQD-COOH.


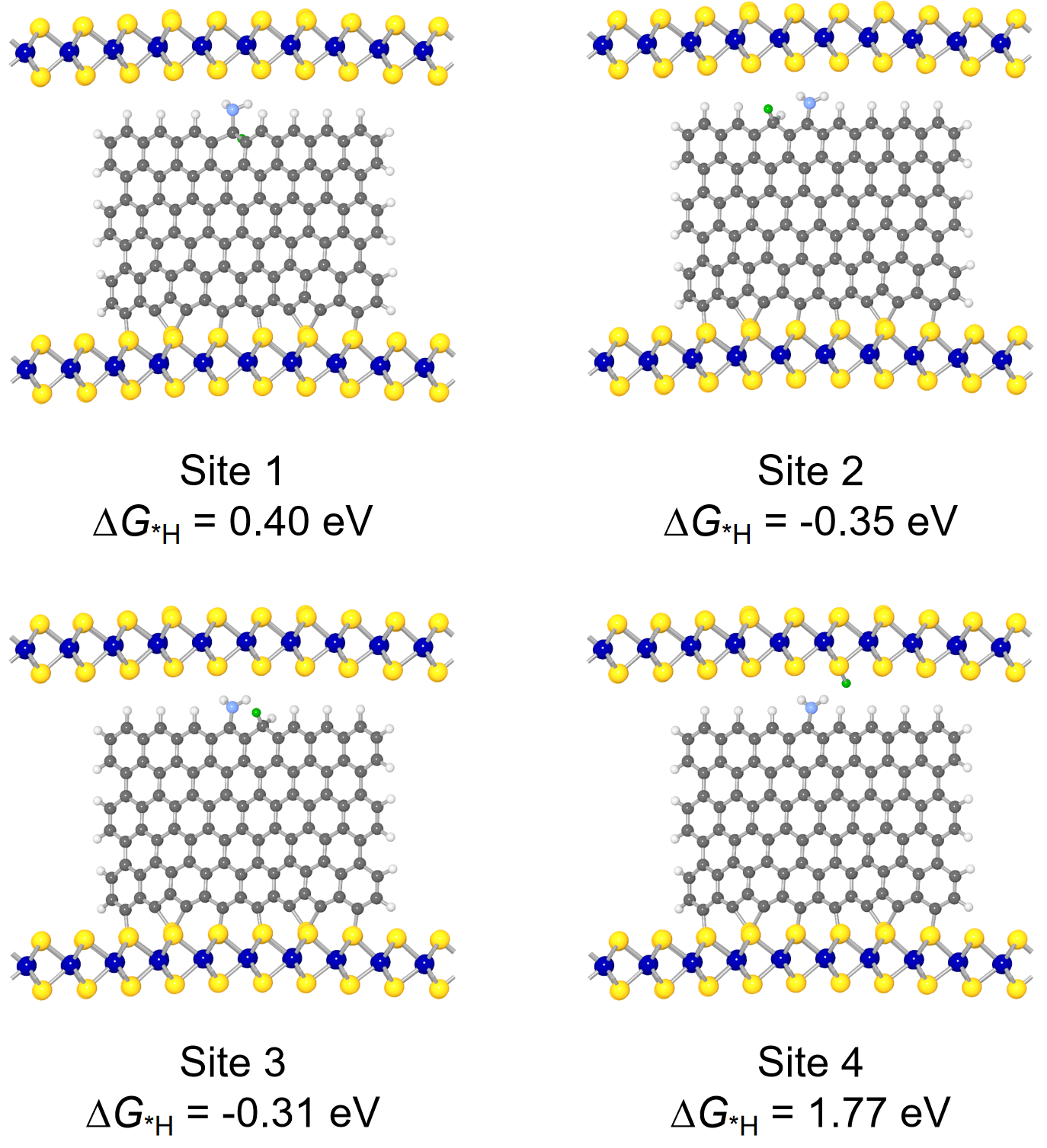


**Fig. S4** Gibbs free energy change (Δ*G*_*H_) of HER process on four sites of ALQD-NH_2_.


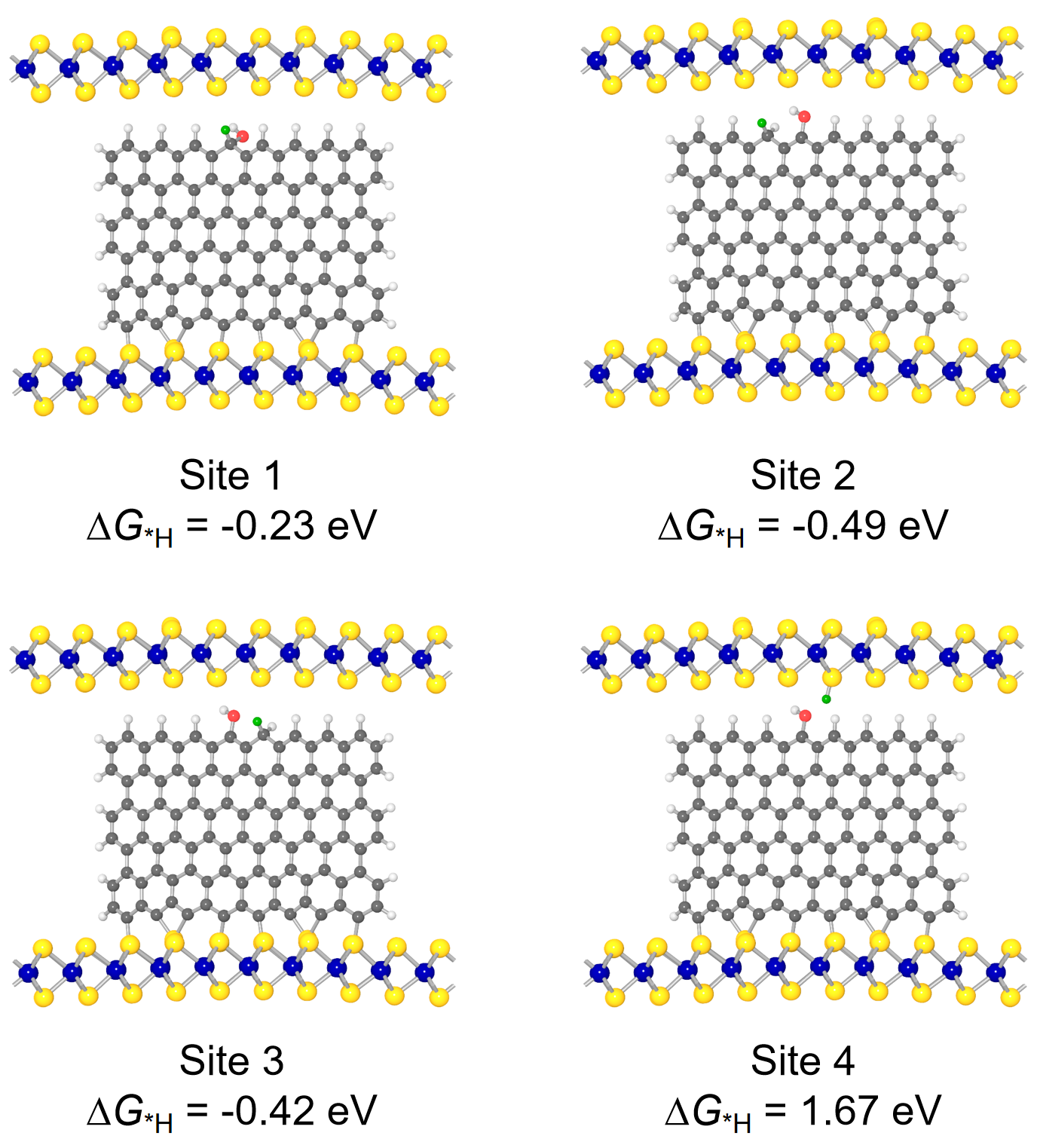


**Fig. S5** Gibbs free energy change (Δ*G*_*H_) of HER process on four sites of ALQD-OH.


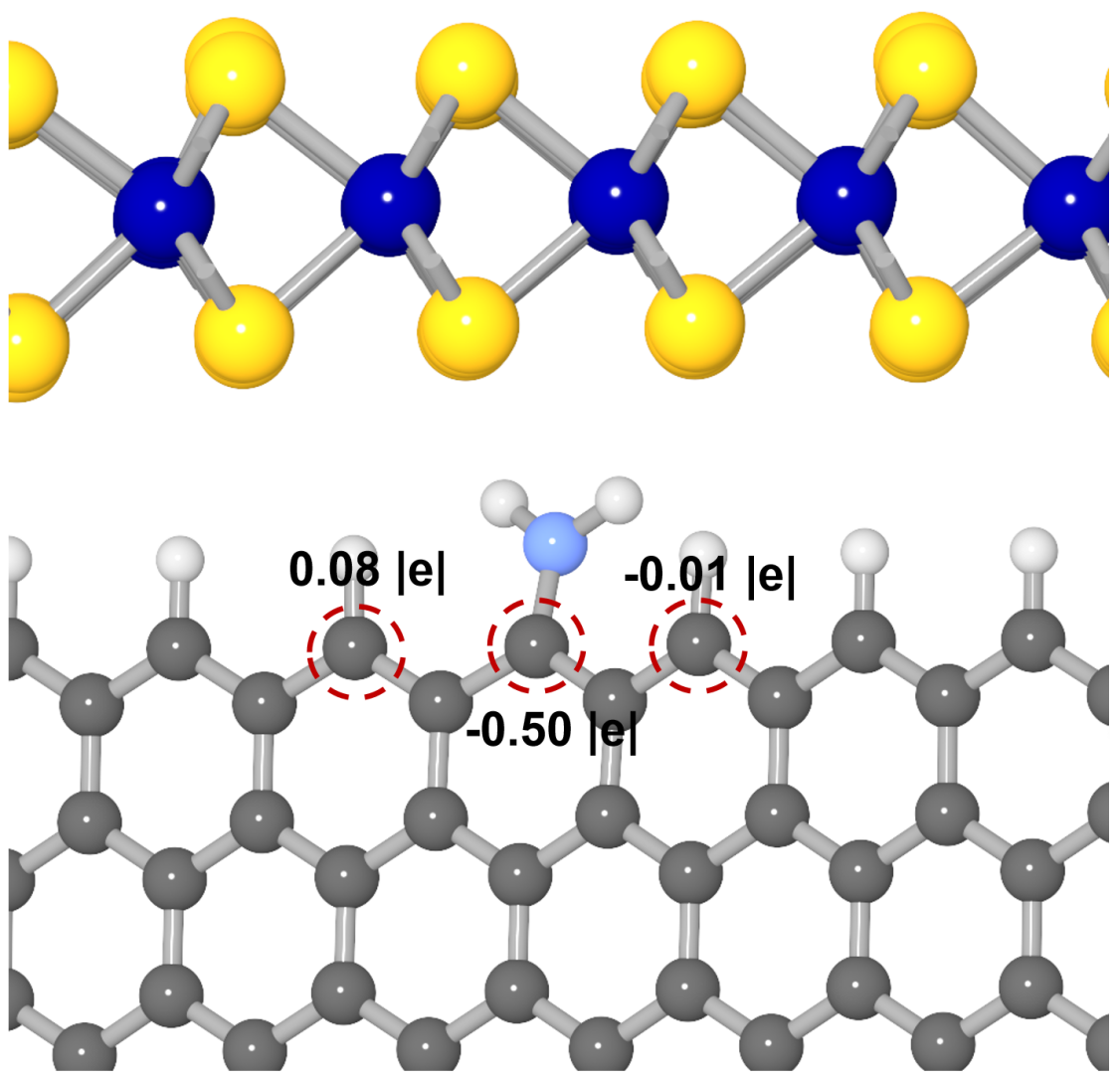


**Fig. S6** Bader charge of C atoms near the functional group and C atoms connecting functional group for ALQD-NH_2_.


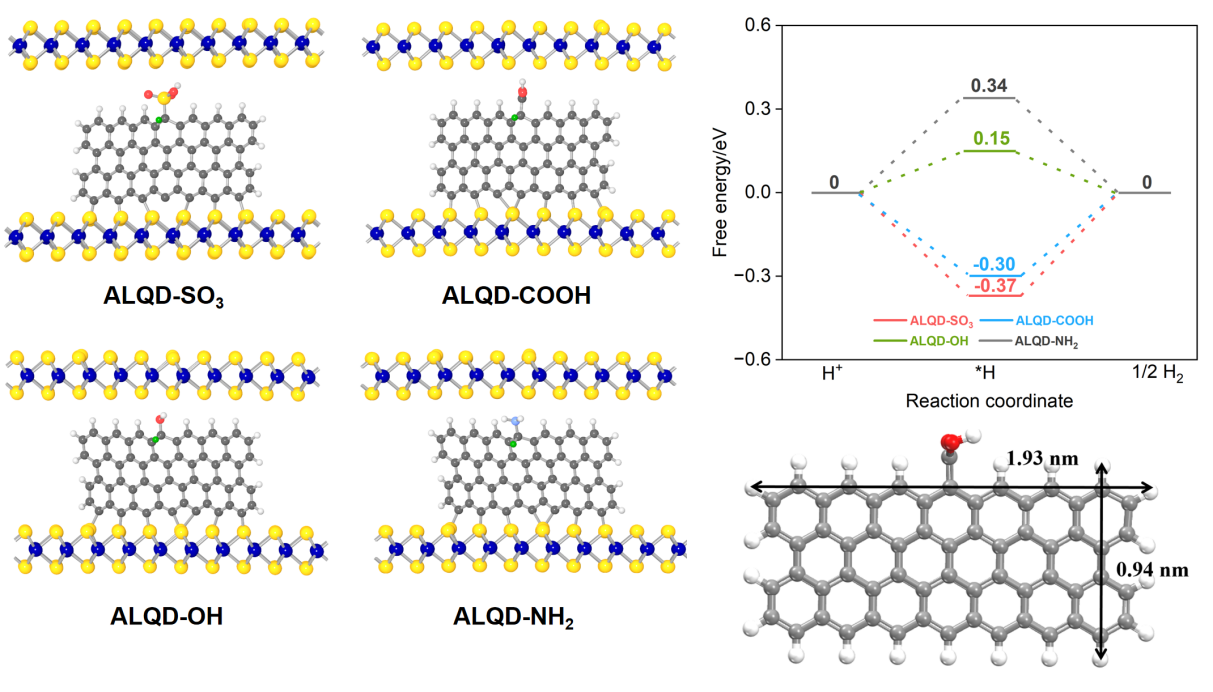


**Fig. S7** Gibbs free energy change (Δ*G*_*H_) of HER process on the C sites connected to the functional group of medium size ALQD.


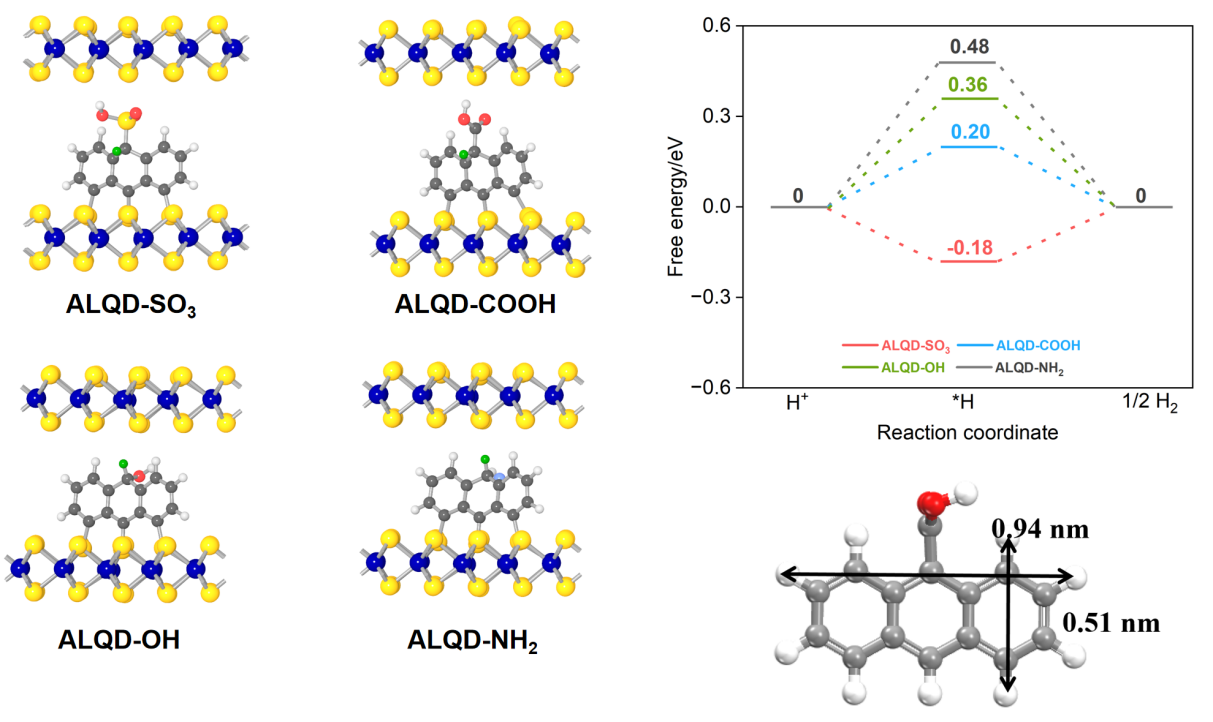


**Fig. S8** Gibbs free energy change (Δ*G*_*H_) of HER process on the C sites connected to the functional group of small size ALQD.

**Fig. S9** The net weight of ALQD-SO_3_ which obtained in one-time synthesis.

**Fig. S10** TEM image of bulk MoS_2_.

**Fig. S11** AFM image of bulk MoS_2_.

**Fig. S12** (a) XPS survey spectra and High-resolution XPS, Mo 3d (b) and S 2p (c) spectra of ALQD-SO_3_ before and after 6 months on standing.

**Fig. S13** Contact angle of water droplets on the surface of ALQD-SO_3_ (a) and bulk MoS_2_ (b).

**Fig. S14** TEM images and corresponding lateral size distributions of SO_3_-GQDs (a), SO_3_-GQDs-1 (b), SO_3_-GQDs-2 (c), and SO_3_-GQDs-3 (d).

**Fig. S15** (a) XRD pattern of SO_3_-GQDs, (b) XRD patterns of SO_3_-GQDs-1, SO_3_-GQDs-2 and SO_3_-GQDs-3.

**Fig. S16** (a) FTIR spectrum of SO_3_-GQDs. (b) FTIR spectra of SO_3_-GQDs-1, SO_3_-GQDs-2 and SO_3_-GQDs-3.

**Fig. S17** XPS survey spectra (a) and high-resolution XPS, C 1s (b), N 1s (c), O 1s (d) and S 2p (e) spectra of SO_3_-GQDs, SO_3_-GQDs-1, SO_3_-GQDs-2 and SO_3_-GQDs-3.

**Fig. S18** TEM images of ALQD-SO_3_-1 (a), ALQD-SO_3_-2 (b) and ALQD-SO_3_-3 (c).

**Fig. S19** AFM images of ALQD-SO_3_-1 (a), ALQD-SO_3_-2 (b) and ALQD-SO_3_-3 (c).

**Fig. S20** XPS survey (a), and high-resolution XPS Mo 3d (b), S 2p (c), C 1s (d) and O 1s (e) spectra of ALQD-SO_3_-1, ALQD-SO_3_-2, and ALQD-SO_3_-3.

**Fig. S21** Raman spectra of ALQD-SO_3_-1, ALQD-SO_3_-2 and ALQD-SO_3_-3.

**Fig. S22** XRD patterns of ALQD-SO_3_-1, ALQD-SO_3_-2 and ALQD-SO_3_-3.

**Fig. S23** Contact angle of water droplets on the surface of ALQD-SO_3_-1 (a), ALQD-SO_3_-2 (b) and ALQD-SO_3_-3 (c).

**Fig. S24** LSV curve (a) and corresponding Tafel plots (b) for glassy carbon electrodes deposited with functionalized, ALQD-SO_3_-1, ALQD-SO_3_-2 and ALQD-SO_3_-3. LSVs are taken at 10 mV/s in 0.5 M H_2_SO_4_ with a Hg/Hg_2_Cl_2_ reference electrode and vitreous carbon counter electrode. Pt and glassy carbon electrodes for HER are included in (a) as a reference.

**Fig. S25** TEM images and corresponding lateral size distributions of GQDs-COOH (a) and GQDs-OH (b).

**Fig. S26** XRD patterns of GQDs-COOH and GQDs-OH.

**Fig. S27** XPS survey spectra (a) and high-resolution XPS, C 1s (b), N 1s (c) and O 1s (d) spectra of GQDs-COOH and GQDs-OH.

**Fig. S28** XRD patterns of ALQD-COOH and ALQD-OH.

**Fig. S29** Raman spectra of ALQD-COOH and ALQD-OH.

**Fig. S30** XPS survey (a), and high-resolution XPS Mo 3d (b), S 2p (c), C 1s (d) and O 1s (e) spectra of ALQD-COOH and ALQD-OH.

**Fig. S31** Contact angle of water droplets on the surface of ALQD-COOH (a) and ALQD-OH (b).

**Fig. S32** XPS survey spectra (a) and high-resolution XPS, C 1s (b), N 1s (c) and O 1s (d) spectra of GQDs-NH_2_, XPS survey (e), and high-resolution XPS Mo 3d (f) and S 2p (g) spectra of ALQD-NH_2_ and bulk MoS_2_.

**Fig. S33** XRD patterns of GQDs-NH_2_ (a) and ALQD-NH_2_ (b).

**Fig. S34** (a) TEM image and corresponding lateral size distribution of GQDs-NH_2_. (b) TEM image of ALQD-NH_2_.

**Fig. S35** AFM image of ALQD-NH_2_.

**Fig. S36** LSV curve (a) and corresponding Tafel slope (b) of ALQD-NH_2_. LSVs are taken at 10 mV/s in 0.5 M H_2_SO_4_ with a Hg/Hg_2_Cl_2_ reference electrode and vitreous carbon counter electrode. Pt and glassy carbon electrodes for HER are included in (a) as a reference.

**Table S1** Element content of ALQD-SO_3_, ALQD-SO_3_-1, ALQD-SO_3_-2, ALQD-SO_3_-3, ALQD-COOH, ALQD-OH, ALQD-NH_2_ and bulk MoS_2_.

|  | **Mo 3d**  (at.%) | **S 2p**  (at.%) | **-SO_3_H**  (at.%) | **C 1s**  (at.%) | **N 1s**  (at.%) | **-NH_2_**  (at.%) | **O 1s**  (at.%) | **C=O**  (at.%) | **C-O**  (at.%) |
| --- | --- | --- | --- | --- | --- | --- | --- | --- | --- |
| **ALQD-SO_3_** | 19.35 | 26.17 | 3.8 | 31.74 | 2.81 | - | 19.94 | - | - |
| **ALQD-SO_3_-1** | 16.91 | 23.62 | 3.56 | 35.97 | 4.56 | 0.88 | 18.94 | - | - |
| **ALQD-SO_3_-2** | 17.17 | 28.03 | 3.11 | 32.82 | 3.72 | 0.72 | 18.26 | - | - |
| **ALQD-SO_3_-3** | 16.5 | 27.54 | 2.65 | 33.4 | 2.08 | 0.51 | 20.48 | - | - |
| **ALQD-COOH** | 14.77 | 28.2 | - | 25.78 | 5.0 | - | 26.23 | 23.0 | 3.22 |
| **ALQD-OH** | 19.35 | 36.3 | - | 21.27 | 6.43 | - | 16.65 | 0.64 | 16.01 |
| **ALQD-NH_2_** | 17.29 | 32.34 | - | 24.8 | 4.95 | 0.66 | 20.62 | - | - |
| **bulk MoS_2_** | 38.59 | 61.41 | - | - | - | - | - | - | - |

**Table S2** Element content of SO_3_-GQDs, SO_3_-GQDs-1, SO_3_-GQDs-2, SO_3_-GQDs-3.

|  | **C 1s**  (at.%) | **N 1s**  (at.%) | **O 1s**  (at.%) | **S 2p**  (at.%) | **-SO_3_H**  (at.%) |
| --- | --- | --- | --- | --- | --- |
| **SO_3_-GQDs** | 52.2 | 3.84 | 36.47 | 7.49 | 7.49 |
| **SO_3_-GQDs-1** | 70.64 | 5.41 | 19.56 | 4.39 | 4.39 |
| **SO_3_-GQDs-2** | 66.14 | 8.93 | 20.83 | 4.1 | 4.1 |
| **SO_3_-GQDs-3** | 72.52 | 4.15 | 21.18 | 2.15 | 2.15 |

**Table S3** Element content of COOH-GQDs, OH-GQDs, NH_2_-GQDs.

|  | **C 1s**  (at.%) | **N 1s**  (at.%) | **O 1s**  (at.%) | **C=O**  (at.%) | **C-O**  (at.%) |
| --- | --- | --- | --- | --- | --- |
| **COOH-GQDs** | 69.35 | 3.64 | 27.01 | 22.69 | 4.31 |
| **OH-GQDs** | 75.73 | 6.96 | 17.31 | 7.42 | 9.89 |
| **NH_2_-GQDs** | 65.82 | 8.55 | 25.63 | - | - |
